# *TGFβ1* overexpression is associated with improved survival and low tumor cell proliferation in patients with early-stage pancreatic ductal adenocarcinoma

**DOI:** 10.18632/oncotarget.13533

**Published:** 2016-11-23

**Authors:** Evan S. Glazer, Eric Welsh, Jose M. Pimiento, Jamie K. Teer, Mokenge P. Malafa

**Affiliations:** ^1^ Department of Gastrointestinal Oncology, H. Lee Moffitt Cancer Center and Research Institute, Tampa, FL, USA; ^2^ Department of Biostatistics and Bioinformatics, H. Lee Moffitt Cancer Center and Research Institute, Tampa, FL, USA; ^3^ Department of Surgery, University of Tennessee Health Sciences Center, Memphis, TN, USA

**Keywords:** pancreatic carcinoma, TGF-β, Ki-67, MKI67

## Abstract

The role of transforming growth factor beta-type-1 (TGFβ1) in pancreatic ductal adenocarcinoma (PDAC) progression is stage-dependent. We hypothesized that *TGFβ1* expression is associated with survival and proliferation markers in patients with early-stage PDAC. We acquired clinicopathologic, treatment, and mRNA expression data from The Cancer Genome Atlas data set for 106 patients identified with stage I/II PDAC who underwent pancreaticoduodenectomy. Patients were categorized as high expression when mRNA expression was ≥75th percentile for each gene. Average log_2_ mRNA expression of *TGFβ1* in patients with high expression was 11.6 ± 0.2 and 10.5 ± 0.6 in patients with low expression (*P*<0.001). Low *TGFβ1* expression is associated with shorter median survival compared with high *TGFβ1* expression (17 versus at least 60 months; *P*=0.005). Patients with tumors demonstrating high *MKI67* (the gene encoding Ki-67) expression have shorter median survival versus those with lower*MKI67* expression (16 versus 20 months; *P*=0.026). *TGFβ1* and *MKI67* are inversely associated (*P*=0.009). On multivariate analysis, improved survival is associated with *TGFβ1* overexpression (*P*=0.017), adjuvant chemotherapy (*P*=0.001), and adjuvant radiotherapy (*P*=0.017), whereas positive surgical margins are associated with worse survival (*P*=0.002). In patients who undergo pancreaticoduodenectomy for PDAC, high *TGFβ1* expression may counteract the worse survival associated with high proliferation.

## INTRODUCTION

The precise role of transforming growth factor beta type 1 (TGFβ1) in the progression of pancreatic ductal adenocarcinoma (PDAC) is unknown, and its relation to survival is context dependent [1, 2]. This is referred to as the TGFβ paradox, where *TGFβ* expression in early tumors (AJCC stage I and II) is a favorable characteristic but a detrimental characteristic in advanced-stage III or IV disease [3].

TGFβ is a complex signaling molecule that exists in 3 isoforms and as part of large family of polypeptides [4]. Although it is critical for growth arrest, it is also a growth factor responsible for differentiation in embryonic progenitor cells [5]. Among other signaling cascades, the canonical pathway for activated TGFβ receptors leads to downstream serine phosphorylation of SMAD transcription factors [6]. Important in PDAC, TGFβ signaling induces peritumoral fibrosis by myofibroblast differentiation when exposed to radiation [7] or by increased collagen production in the setting of chronic pancreatitis [8].

Although the role of TGFβ has been better delineated in late-stage PDAC, its role in early-stage PDAC is unclear. In late-stage PDAC, targeted therapeutics against the TGFβ pathway are undergoing clinical trials as data are more mature in this area [9] and suggest that angiogenesis is critical to PDAC tumorigenesis [3, 10]. There are conflicting data in the literature regarding the role of TGFβ in patients with early-stage PDAC. Some evidence has suggested that early-stage tumors are similar to late-stage tumors in terms of a proangiogenic environment [11]. However, other data have suggested that TGFβ blocks tumor cell proliferation [12]. Due to these conflicting data, we investigated the association between *TGFB*1 and RNA expression of the proliferation marker Ki-67 in PDAC.

Because of its complex signaling cascade, it has been suggested that *TGFβ* is both a proto-oncogene and tumor suppressor gene, depending on the context [6, 13]. Work by Zhang et al. have demonstrated that TGFβ1 overexpression inhibits cell growth via SMAD3 phosphorylation, hence behaving as a tumor suppressor [3]. Likewise, that group also provided limited data that TGFβ1 overexpression results in SMAD7 activation, which leads to β-catenin/VEGF-A signaling and tumor neo-angiogenesis. Others have demonstrated similar oncogenic behavior of TGFβ, especially in the context of proliferation and inflammation [2, 10].

A potential mechanism for TGFβ1*-*mediated carcinogenesis is an overwhelming, proliferative peritumoral desmoplastic response [14]. In a non-malignant environment, collagen production by fibroblasts is mediated by TGFβ1 signaling [7, 15, 16]. In the malignant setting, pre-clinical and *in vivo* data have suggested that collagen deposition by pancreatic stellate cells and pancreatic carcinoma cells both lead to a pro-carcinogenic desmoplastic response [17–19]. In addition, increased proliferation as measured by Ki-67 expression in PDAC cancer cells is associated with therapeutic changes in animal models and early recurrence in retrospective human studies [20–22]. Interestingly, an mRNA transcript leading to type I collagen production, *COL1A2,* has been associated with proliferative events and markers of aggressive tumor biology, including epithelial to mesenchymal transition [8]. Another group recently demonstrated that the same specific mRNA transcript (*COL1A2*) may be a serum biomarker for pancreatic carcinoma [19].

We hypothesized that survival in patients with resected early-stage PDAC is associated with *TGFβ1* and *MKI67* expression (the gene encoding the proliferation marker Ki-67). To control for unknown features of cancer biology, we utilized a strict inclusion/exclusion criteria to identify a relatively homogenous patient population from The Cancer Genome Atlas (TCGA) with stage I and II PDAC who underwent pancreaticoduodenectomy.

## RESULTS

### Early pancreatic ductal adenocarcinoma in the TCGA data set

Our TCGA data set consisted of 175 tumor samples with pancreatic malignancies. Five of these patients demonstrated histologies not consistent with “typical” PDAC to include neuroendocrine and mucinous colloid carcinomas. Likewise, a minority of patients underwent distal pancreatectomy, total pancreatectomy, or other pancreatic resection (n = 38) and 10 patients had stage III or IV disease.

After applying the inclusion and exclusion criteria, 106 patients were identified for analyses. Of these patients, the average age was 65 ± 11 years, 58% of patients were male, and 91% were white. The median follow-up time was 15 months (range, 0.3-75 months). Seven patients (7%) had a documented family history of PDAC. Only 1 patient received neoadjuvant therapy. In our patient group, 13%, 58%, and 28% of tumors were histologically grade I, II, and III/IV, respectively. The mean number of lymph nodes examined was 18 ± 8 per patient with an average of 3 ± 3 positive lymph nodes per patient. Overall, 60 patients (57%) received a margin-negative (R0) resection, while 56 patients received margin-positive resections (R1 or R2).

### TGFβ1 expression in early PDAC from the TCGA

**Figure 1:**
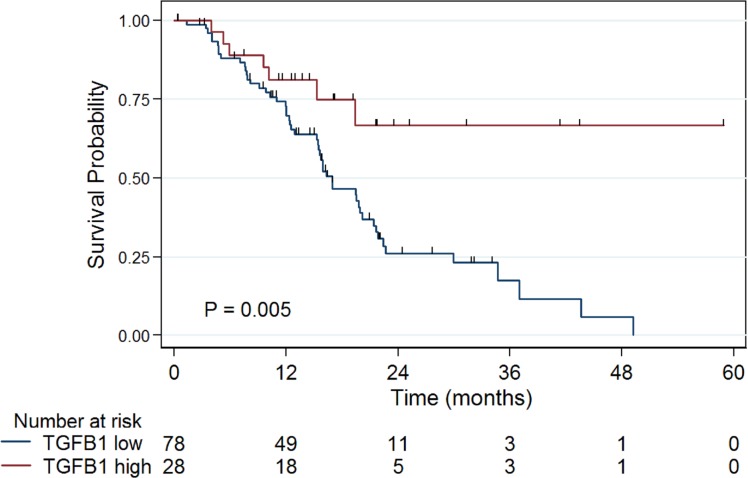
Graphical representation of the log_2_ mRNA levels of *TGFβ1* A. *MKI67* B. *COL1A2* C

The average expression of *TGFβ1* in the top quartile (high expression group) was 11.6 ± 0.2, whereas it was 10.5 ± 0.6 in patients with low expression (*P* < 0.001, Figure 1A). mRNA expression of *MKI67* was 11.2 ± 0.4 in the high expression group and 9.8 ± 0.8 in the low expression group (Figure 1B, P<0.001). Finally, log_2_ mRNA expression of *COL1A2* was 17.5 ± 0.5 in the high expression group and 15.7 ± 1.1 in the low expression group (P<0.001, Figure 1C).

### High *TGFβ1* expression is associated with improved overall survival

**Figure 2:**
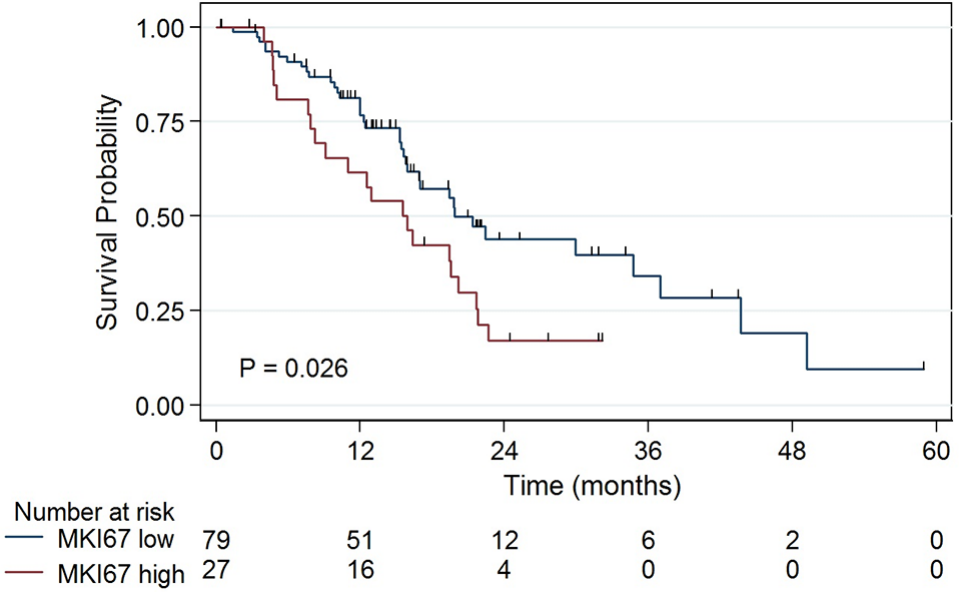
Patients with tumors demonstrating TGFβ1 expression ≥75^th^ percentile (high TGFβ1) have longer median survival than other patients (> 60 vs 17 months, *P* = 0.005)

The clinical and treatment characteristics for patients with tumors expressing high or low levels of *TGFβ1* were similar between the two groups, with the exception of radiotherapy (Table 1). Low *TGFβ1* expression was associated with median survival of 17 months, whereas median survival was not reached for the high *TGFβ1* expression group (at least 60 months, Figure 2; *P* = 0.007). All long-term survivors (> 40 months) were in the high *TGFβ1* expression group. The univaraiate Cox proportion hazard ratio (HR) for high *TGFβ1* group was 0.35 (*P* = 0.009; Table 2). Finally, the mean log_2_ expression level of *TGFβ1* in patients with a family history of PDAC was lower than in patients without a family history of PDAC (10.18 versus 10.84; *P* = 0.02).

**Table 1:** Patient and treatment characteristics

Characteristic	TGFβ1 Low	TGFβ1 High	*P* Value
	n = 78	n = 28	
Age, mean ± SD	65 ± 11 years	66 ± 10 years	0.61
Male, n (%)	46 (58%)	16 (59%)	0.92
AJCC Stage			0.96
T1-2	8 (10%)	5 (18%)	
T3	70 (90%)	23 (82%)	
N1	63 (80%)	21 (78%)	
Family history of pancreatic cancer, n (%)	7 (9%)	0 (0%)	0.11
High-grade tumor, n (%)	22 (28%)	8 (29%)	0.97
Positive surgical margin, n (%)	34 (44%)	12 (43%)	0.9
Radiotherapy, n (%)	17 (22%)	15 (57%)	**0.003**
Chemotherapy, n (%)	55 (71%)	21 (75%)	0.65

**Table 2:** Characteristics associated with overall survival

Variable	Univariate Cox HR	*P* Value	Multivariate Cox HR	*P* Value
Age, per year	1.03	0.03	1.01	0.428
Male gender	0.67	0.13		
Family history	2.26	0.06	**3.73**	**0.02**
Positive lymph nodes	2.61	0.019	**3.68**	**0.013**
Positive surgical margin	2.3	0.003	**3.37**	**0.002**
High-grade tumor	1.67	0.07	1.62	0.179
Chemotherapy	0.39	0.001	**0.30**	**0.001**
Radiotherapy	0.36	0.003	**0.38**	**0.017**
High *TGFβ1* expression	0.35	0.009	**0.29**	**0.017**
High *MKI67* expression	1.85	0.028	1.37	0.373
High *COL1A2* expression	0.45	0.02	0.77	0.567

Interestingly, high mRNA expression of SMAD3, SMAD4, and TGFβR1 were not associated with survival (P=0.87, 0.53, and 0.14, respectively). Likewise, there was no association between the mRNA expressions of these genes with expression of *TGFβ1* (each *P* > 0.1).

### Increased proliferation is associated with worse survival

**Figure 3:**
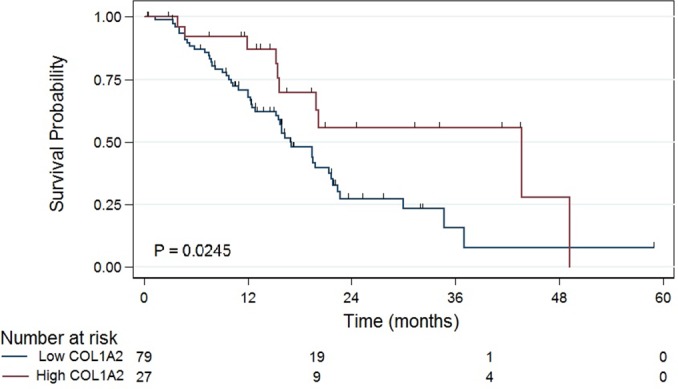
Patients with tumors demonstrating *MKI67* expression ≥75^th^ percentile have shorter median survival than patients with tumors demonstrating lower *MKI67* expression (16 vs 20 months, *P* = 0.026)

Because Ki-67 is an established marker for cell proliferation in cancer, we investigated its mRNA expression in PDAC. Patients with tumors demonstrating *MKI67* expression ≥75th percentile have shorter median survival versus patients with tumors demonstrating lower *MKI67* expression (16 months versus 20 months; *P* = 0.026, Figure 3). Interestingly, patients in the high *MKI67* expression group showed lower levels of *TGFβ1* expression compared with patients in the low *MKI67* expression group (0.07 versus 0.33; *P* = 0.009). Furthermore, 32% of patients with low tumor *TGFβ1* expression have high *MKI67* expression, whereas only 7% of patients in the high *TGFβ1* expression group have high *MKI67* expression (*P* = 0.009).

Because there was correlation between *TGFβ1* and *MKI67* expression, we investigated survival in patients by gene expression status (Table 2). The median survival in the low *TGFβ1*/low *MKI67* expression group was 17 months, whereas patients in the high/high group demonstrated a median survival of 4 months. In the low *TGFβ1*/high *MKI67* expression group, the median survival was 16 months, whereas it was greater than 60 months in the high *TGFβ1*/low *MKI67* expression group (overall *P* = 0.015). All long-term survivors were in the high/low group.

On multivariate Cox regression analyses, high *TGFβ1* expression remained associated with longer survival (Cox HR = 0.29; *P* = 0.017). Neither *MKI67* expression nor *COL1A2* expression was independently associated with survival on multivariate Cox regression (Table 2). However, a positive surgical margin remained not only statistically associated with worse survival on multivariate analysis, but it had one of the largest associations with overall survival except for high *TGFβ1* expression and a family history of PDAC (Table 2). Importantly, whereas high *TGFβ1* expression is associated with radiotherapy (Table 1) and radiotherapy was associated with survival on univariate analysis (Table 2), on multivariate analysis *TGFβ1* expression remained statistically significantly associated with longer survival and radiotherapy showed less of an overall effect on survival than high *TGFβ1* expression (Cox HR = 0.38, *P* = 0.017).

**Figure 4:**
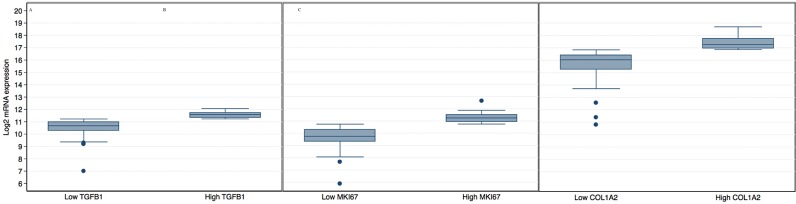
High expression of *COL1A2* is associated with prolonged survival in patients with resected early-stage pancreatic carcinoma (*P* = 0.0245)

Overexpression of *COL1A2*, a component of collagen production, is associated with high *TGFβ1* expression. We found that 51% of tumors in the high *TGFβ1* expression group had high *COL1A2* expression, whereas only 24% of tumors in the low *TGFβ1* expression group had high *COL1A2* expression (*P* < 0.001). Furthermore, median survival was significantly lower in the low *COL1A2* expression group (Figure 4; 17 versus 44 months; *P* = 0.02). In the low *TGFβ1* expression group, high *COL1A2* expression was associated with improved survival (Cox HR = 0.39; *P* = 0.03). There was no association between *MKI67* and *COL1A2* expression (data not shown; *P* = 0.3).

Of the 106 patients, the overall Kaplan-Meier median survival was 19 months. On univariate analyses, age, positive surgical margins, chemotherapy, radiotherapy, high *TGFβ1* expression, high *MKI67* expression, high *COL1A2* expression, and positive lymph node spread were all statistically associated with survival (Table 2). Radiotherapy was significantly associated with longer survival in margin-positive (R1 or R2) resections (Cox HR = 0.3; *P* = 0.01), whereas radiotherapy was not significantly associated with longer survival in margin-negative (R0) resection (Cox HR = 0.4; *P* = 0.07).

## DISCUSSION

The TGFβ1 signaling pathway activates transcription factors intimately involved in many normal and benign functions that, when commandeered by PDAC, permit the cancer to be refractory to standard therapy. For example, fibrosis, a critical response to wound healing, also provides PDAC with a mechanical barrier to chemotherapeutic delivery [23, 24]. Adding to the complexity (and perhaps the paradox), work by Jones et al identified over 30 distinct mutations in the TGFβ1 signaling pathway in 24 pancreatic carcinoma tumors, with 100% of tumors containing at least 1 mutation [25]. While this suggests that the TGFβ1 signaling pathway may be extremely important in PDAC carcinogenesis, it also demonstrates that the TGFβ1 signaling pathway may be far more complex than other common mutated pathways.

In this study, our data suggest that the TGFβ1 paradox may be related to proliferation in patients who undergo pancreaticoduodenectomy for PDAC. High *TGFβ1* expression may be able to ameliorate the decreased survival associated with highly proliferative tumors that have high levels of Ki-67 mRNA. Alternatively, high levels of Ki-67 may overwhelm tumor suppressor effects of TGFβ1. Importantly, in the multivariate analysis, *MKI67* expression was no longer significant when analyzed in conjunction with the other variables in the model, specifically *TGFβ1* and radiotherapy, a treatment that targets proliferating cells. High *TGFβ1* expression remained statistically significant and has the greatest association with survival except for family history of PDAC and positive lymph node disease (Table 2). While PDAC family history is a non-modifiable factor, positive lymph node disease is the definition of aggressive tumor biology on the macroscopic level. Further investigations are needed to delineate the signaling interactions between other proliferation markers and the TGFβ pathway, which may help further unravel the paradox. Finally, the role of Ki-67 in predicting radiosensitivity needs to be elucidated in PDAC.

We investigated the role of *COL1A2* expression by PDAC cells since collagen deposition is often a key component of the desmoplastic response, a component of epithelial to mesenchymal transition, and this specific mRNA transcript may serve as a biomarker for PDAC [8, 19, 26]. Although it has been demonstrated that the peritumoral desmoplasia is created by pancreatic stellate cells [17, 24], we did not identify any evidence in the literature of a relationship between *COL1A2* and survival in patients with PDAC. The data here demonstrated a survival advantage associated with high *COL1A2* expression in patients with PDAC, but, importantly, this was not significant on multivariate analysis where other patients and tumor characteristics dominated the survival model. Most patients in the high *TGFβ1* expression group had high *COL1A2* expression and low *MKI67* expression, which was not seen in the low *TGFβ1* expression group, suggesting the possibility of other interactions not currently investigated. In the low *TGFβ1* expression group, high *COL1A2* expression was statistically associated with improved survival. It is unclear whether PDAC cells are actually producing collagen and creating a desmoplastic response or whether this is an effect (autocrine or otherwise) to aberrant TGFβ1 signaling pathways.

While analyzing a large data set such as the TCGA is quite useful to investigate the interaction of gene expression and clinical data, there are some significant limitations. The major limitation of this analysis is the inability to interrogate tissue specimens or use patient-derived samples to test hypotheses. Specifically, correlating TGFβ1 serum levels, TGFβ1 tissue levels, and clinical outcomes is a future research target to help exact the relationship between *MKI67* expression and TGFβ1 levels. However, these data can serve to outline future research targets, specifically the interaction between cellular proliferation and TGFβ1 pathways. Furthermore, we attempted to limit the effects of the TGFβ1 paradox by using strict inclusion/exclusion criteria to identify patients with “early” disease. However, there is no method to objectively define this patient population other than the self-fulfilling prophecy of investigating patients with only stage I and II PDAC who went to resection. This is selection bias is difficult to quantify.

In summary, our study demonstrates the potential role of proliferation to help understand the TGFβ1 paradox. Patients with early-stage PDAC have longer median survival when *TGFβ1* expression is high, which may be due to limited cellular proliferation. Further work is needed to delineate the mechanism of this interaction, investigate it in patients with advanced PDAC, and identify opportunities to modulate the intra- and extracellular TGFβ1 signaling cascade to improve survival and decrease PDAC proliferation.

## PATIENTS AND METHODS

### Patients and data acquisition

Data from TCGA were downloaded on February 5, 2016. Clinical data, tumor DNA sequence, and mRNA expression data were acquired from the TCGA data set from the data access committee. Except for a single patient, this represents an untreated patient population with PDAC who went directly to surgery for resection. After resection, tumor specimens were dissected from surrounding stroma. mRNA was sequenced with next generation sequencing, usually on the Illuminia platform, as part of the TCGA network with quality controls in place at multiple steps. The TCGA project has been well-described elsewhere [27], but briefly it is a consortium of numerous institutions collaborating to collect, analyze, and integrate data at the genomic, RNA, proteomic, and clinical level for a number of human malignancies.

### Inclusion and exclusion criteria

Inclusion criteria were complete data available, pancreatic adenocarcinoma or ductal carcinoma histology, American Joint Committee on Cancer (AJCC) stage I or II, and underwent pancreaticoduodenectomy. Patients with stage III or IV disease based on the 7th edition of the AJCC staging system were excluded.

### Expression and gene analyses

Patient clinical data (Level 2.0.46.0) and RNASeqV2 normalized gene expression data (Level 3.1.8.0) were downloaded from the TCGA data portal (https://tcga-data.nci.nih.gov/) open access HTTP directory. Gene expression data were further normalized against the median sample using IRON v2.1.6 (iron_generic --rnaseq --unlog) to correct for the remaining minor differences in dynamic range between samples [28]. High gene expression was based on ≥ 75th percentile log_2_ mRNA expression value for each gene. Low gene expression was based on values < 75th percentile log_2_ mRNA expression value for each gene. We specifically investigated the relationship among *TGFβ1*, *MKI67*, and *COL1A2* and survival in patients with resected PDAC in the head of the pancreas and staged as I or II.

### Statistical analyses

Data analyses were performed with STATA 13 (StataCorp LP, College Station, TX). Significance was set to α = 0.05. Differences in variables between the two groups were investigated with Student *t* test or χ^2^ as appropriate. Overall survival was investigated with the Kaplan-Meier method utilizing the log-rank test. Censoring occurred for days to last follow-up with the number of patients at risk listed for each time point below the graphs. Variables associated with survival were investigated with univariate and multivariate Cox regression analyses with the proportional hazard ratio (HR). The multivariate survival model was based on clinical and gene expression data as described below utilizing a cut-off of univariate *P* = 0.1 to be included in the model.
